# Genome-Wide Studies of Histone Demethylation Catalysed by the Fission Yeast Homologues of Mammalian LSD1

**DOI:** 10.1371/journal.pone.0000386

**Published:** 2007-04-18

**Authors:** Michael Opel, David Lando, Carolina Bonilla, Sarah C. Trewick, Abdelhalim Boukaba, Julian Walfridsson, James Cauwood, Petra J.H. Werler, Antony M. Carr, Tony Kouzarides, Natalia V. Murzina, Robin C. Allshire, Karl Ekwall, Ernest D. Laue

**Affiliations:** 1 Department of Biochemistry, University of Cambridge, Cambridge, United Kingdom; 2 Gurdon Institute and Department of Pathology, Cambridge, United Kingdom; 3 Department of Biosciences/School of Life Science, Karolinska Institutet, University College Sodertorn, Huddinge, Sweden; 4 Wellcome Trust Centre for Cell Biology, The University of Edinburgh, Edinburgh, United Kingdom; 5 Genome Damage and Stability Centre, School of Biological Sciences, University of Sussex, Falmer, Sussex, United Kingdom; Centre de Regulació Genòmica, Spain

## Abstract

In order to gain a more global view of the activity of histone demethylases, we report here genome-wide studies of the fission yeast SWIRM and polyamine oxidase (PAO) domain homologues of mammalian LSD1. Consistent with previous work we find that the two *S. pombe* proteins, which we name Swm1 and Swm2 (after SWIRM1 and SWIRM2), associate together in a complex. However, we find that this complex specifically demethylates lysine 9 in histone H3 (H3K9) and both up- and down-regulates expression of different groups of genes. Using chromatin-immunoprecipitation, to isolate fragments of chromatin containing either H3K4me2 or H3K9me2, and DNA microarray analysis (ChIP-chip), we have studied genome-wide changes in patterns of histone methylation, and their correlation with gene expression, upon deletion of the *swm1^+^* gene. Using hyper-geometric probability comparisons we uncover genetic links between lysine-specific demethylases, the histone deacetylase Clr6, and the chromatin remodeller Hrp1. The data presented here demonstrate that in fission yeast the SWIRM/PAO domain proteins Swm1 and Swm2 are associated in complexes that can remove methyl groups from lysine 9 methylated histone H3. *In vitro*, we show that bacterially expressed Swm1 also possesses lysine 9 demethylase activity. *In vivo*, loss of Swm1 increases the global levels of both H3K9me2 and H3K4me2. A significant accumulation of H3K4me2 is observed at genes that are up-regulated in a *swm1* deletion strain. In addition, H3K9me2 accumulates at some genes known to be direct Swm1/2 targets that are down-regulated in the *swm1*Δ strain. The *in vivo* data indicate that Swm1 acts in concert with the HDAC Clr6 and the chromatin remodeller Hrp1 to repress gene expression. In addition, our *in vitro* analyses suggest that the H3K9 demethylase activity requires an unidentified post-translational modification to allow it to act. Thus, our results highlight complex interactions between histone demethylase, deacetylase and chromatin remodelling activities in the regulation of gene expression.

## Introduction

Post-translational modifications of histones regulate gene transcription either by recruiting other proteins/complexes or by altering the underlying chromatin structure. Until recently one such modification, lysine methylation, which can either activate or repress gene transcription [for a review see ref. 1], was thought to be irreversible. However, two classes of protein demethylase, that specifically remove methyl groups from lysine, have now been identified [2]–[6]. One of these, represented by lysine-specific demethylase 1 (LSD1), also known as BHC110, is a flavin adenine nucleotide-dependent (FAD) amine oxidase that removes methyl-groups from mono- and di-methylated lysine 4 of histone H3 (H3K4) [2]. LSD1 is a component of various complexes that repress transcription and which often contain HDAC1/2 and CoREST [7]–[10]. Recent studies show that the specificity and activity of the enzyme is modulated by its association with different proteins [11]–[13]. Metzger et al., (2005) [13], have interestingly shown that LSD1 when associated in a complex with the androgen receptor specifically demethylates H3K9 (instead of H3K4). The activity of LSD1 is also modulated by association with a SANT domain from the CoREST protein, which recruits the demethylase to nucleosomal substrates [11]–[12]. In addition, it has also been suggested that demethylation of nucleosomes by the LSD1-CoREST complex is inhibited by BHC80, a PHD domain protein [11], as well as by histone acetylation [12]. These results suggest a model whereby demethylase activity can be targeted in alternative ways to different sites and that it is regulated by other modifications, e.g. acetylation, to coordinate different activities.

## Results and Discussion

### Identification of the members of the Swm complexes

**Figure 1:**
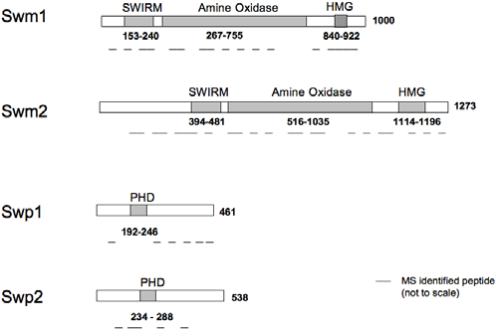
Schematic representation of Swm1/2 complex members: Swm1 (SPBC146.09c), Swm2 (SPAC23E2.02), Swp1 (SPCC4G3.07) and Swp2 (SPAC30D11.08c) are annotated with domain borders as in the SMART sequence analysis database (http://smart.embl-heidelberg.de).

Strains expressing C-terminally TAP-tagged Swm1 and Swm2 (from their endogenous promoters) were used to affinity purify complexes of the two proteins. The associated proteins were subsequently identified by mass spectrometry (MS). The results of the MS analysis are presented in Table S1 in the Supplementary Information and are summarized in Figure 1. In brief, our data confirm the results of Nicolas et al., (2006) [14] and show that the Swm1 complex contains Swm2 and two new PHD domain containing proteins (*S. pombe* DB CDS: SPCC4G3.07 and SPAC30D11.08c), hereafter referred to as Swp1 (**Sw**m associated **P**HD1) and Swp2 (**Sw**m associated **P**HD2). Surprisingly, the purified Swm2 complex contained only Swm1 and Swp1, but not Swp2, suggesting that the Swm proteins may exist in more than one complex. However, in contrast to the results of Nicolas et al., (2006) [14] we did not detect either Hrp1 or SPBPJ758.01 (an RNA recognition motif protein), suggesting that these proteins have weaker affinities or are more transiently associated with the complexes. Alternatively, the use of different tags in the two studies may also explain the discreprency.

### The S. pombe Swm complex demethylates lysine-9 of histone H3

Previous studies have shown that human LSD1 can demethylate either lysine residues 4 or 9 in histone H3, depending on the presence or absence of associated proteins [13]. To determine whether the *S. pombe* Swm complex can also demethylate histones, we carried out a series of demethylation assays using purified TAP-tagged complexes.

Initial assays for histone demethylase activity were attempted using similar methods to those described by Shi et al., (2004) [2], involving MS and synthetic peptide substrates. However, when comparing the detection limits (assuming a similar level of activity to the *E. coli* expressed human LSD1 as a control) we found that we were not able to purify sufficient quantities of the TAP-tagged Swm1 and Swm2 complexes from *S. pombe* to reliably detect demethylase activity in MS assays. We therefore turned to a more sensitive histone demethylase assay, in which purified histone methylases are used to radiolabel histone substrates [5], [15]. In this work we used human Set7, *S. pombe* Clr4 and *S. cerevisiae* Set2 to specifically methylate K4, K9 and K36 in histone H3, either in bulk histones or in polynucleosomes. Because FAD-dependent amine oxidases should demethylate both mono- and di-methylated substrates [2], we used Set7 to monomethylate H3K4 (and not the Set1 complex which leads to trimethylation). We also tried to ensure that the Clr4 reaction (which can also lead to trimethylation of H3K9) was partial – by carrying out the labelling reaction for short periods of time.

**Figure 2:**
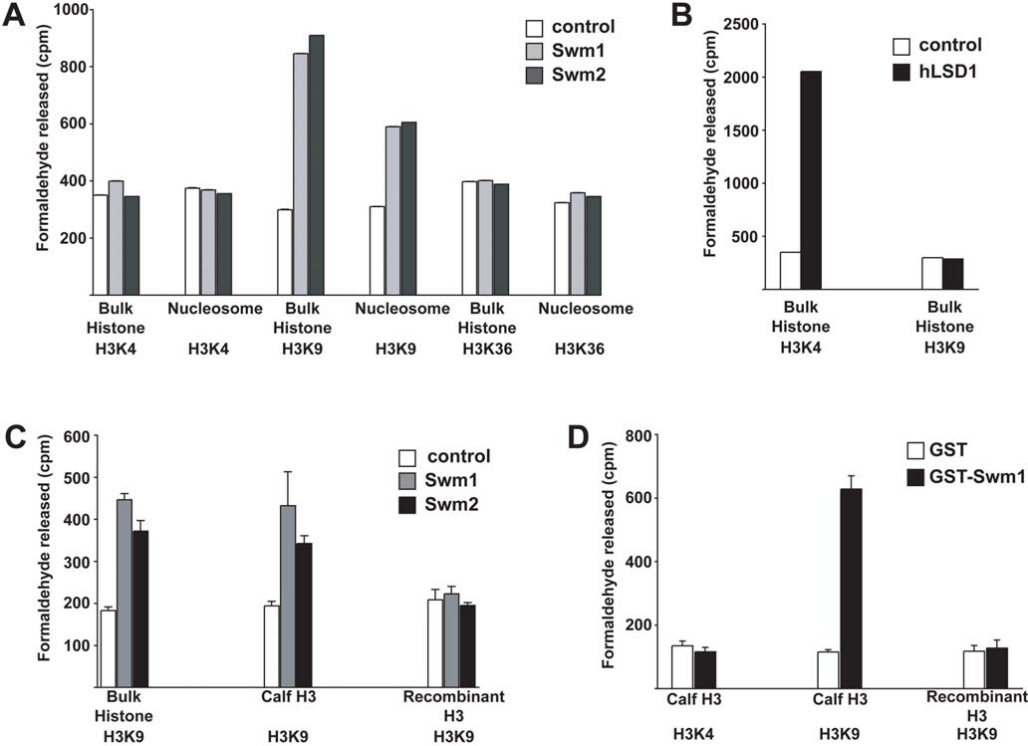
Histone demethylase activity of TAP tagged Swm1 and Swm2 complexes (A&C), recombinant human LSD1 (B), and GST-Swm1 (D), with various methylated histone substrates. The substrates: calf thymus bulk histones (BH), chicken polynucleosomes (Nuc), calf thymus histone H3 (H3) and recombinant H3 (rH3) along with their sites of lysine (K) methylation are indicated below the panels. Control indicates a mock TAP-tag purification from the wild type strain. hLSD1 was recombinant *E. coli* expressed human LSD1 protein and GST-Swm1 was recombinant *E. coli* expressed Swm1.

Interestingly, only methylated H3K9 was found to be a substrate for the *S. pombe* Swm1 and Swm2 complexes (Figure 2a). Similar levels of activity were detected regardless of whether the complex was purified using TAP-tagged Swm1 or Swm2. Moreover, similar levels of activity were found for histone H3 substrates in the form of either purified bulk histones or nucleosomes. By contrast, in the human LSD1 control we observed specific demethylation of H3K4 as previously reported (Figure 2b) [2]. We next tested whether the *S. pombe* Swm1 and Swm2 complexes were able to demethylate K9 in isolated histone H3. While both TAP-tagged complexes were found to have similar activity towards histone H3 purified from calf thymus (in comparison to nucleosomal substrates), neither complex was active on recombinant histone H3 (Figure 2c). It is currently unclear why the *S. pombe* demethylase complex is inactive on recombinant methylated histone H3, but one possibility is that an additional post-translational modification(s) is needed for the complex to effectively recognise its substrate. Finally, to rule out the possibility that a contaminant was responsible for the H3K9 demethylase activity, we demonstrated that recombinant *E. coli* expressed Swm1 has the same pattern of activity/specificity as that of the intact complex (Figure 2d). This experiment demonstrates that Swm1 is a catalytically active subunit. However, because we have not so far been able to express/purify recombinant Swm2, this PAO domain protein may also be catalytically active. Structure-based sequence analysis suggests that Swm2 also has the appropriate FAD-binding and catalytic residues required for demethylase activity (data not shown).

### Swm1 both up- and down-regulates gene expression

To determine if the Swm1 and Swm2 proteins are involved in regulation of gene expression, we constructed strains of *S. pombe* in which either the Swm1 or Swm2 genes were deleted. Consistent with the results obtained by Nicolas et al., (2006) [14], we found that *swm2* is an essential gene and that deletion of *swm1* markedly increased the cell doubling time (data not shown). Thus, gene expression profiling could only be carried out in cells lacking Swm1.

**Figure 3:**
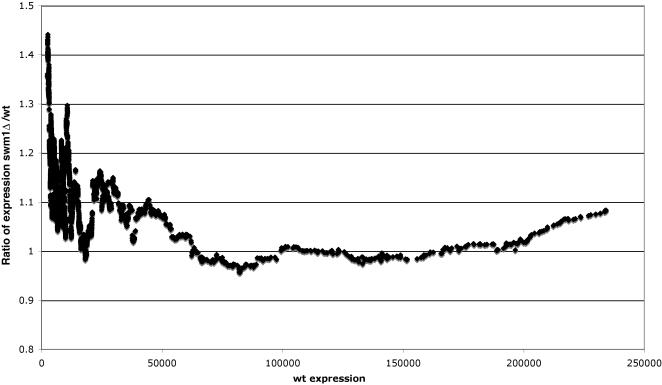
Global analysis of the gene expression profile in *swm1* *vs* wild type cells. A moving average plot (window size = 150 genes, step size = 1 gene) of the median gene expression ratio, *swm1*Δ/wt, plotted as a function of the average transcription levels of 11 wild-type cell cultures in mid-logarithmic growth.

Our global analysis of gene expression shows that somewhat more genes are up-regulated in the *swm1* deletion (*swm1*Δ) strain (265 genes) than are down-regulated (173 genes) (using a 1.5 fold cut-off; see Table S2 in the Supplementary information). In Figure 3, the moving average (calculated over 150 genes) of the *swm1*Δ/wild-type expression ratio *vs* the level of wild-type gene expression is plotted. If the *swm1* deletion had no effect on gene expression one would expect a global moving average ratio of 1.0. As can be seen, however, the *swm1*Δ/wild-type expression ratio is generally high for non-expressed and weakly expressed genes, suggesting that (globally) Swm1 represses these non-expressed and weakly expressed genes.

**Figure 4:**
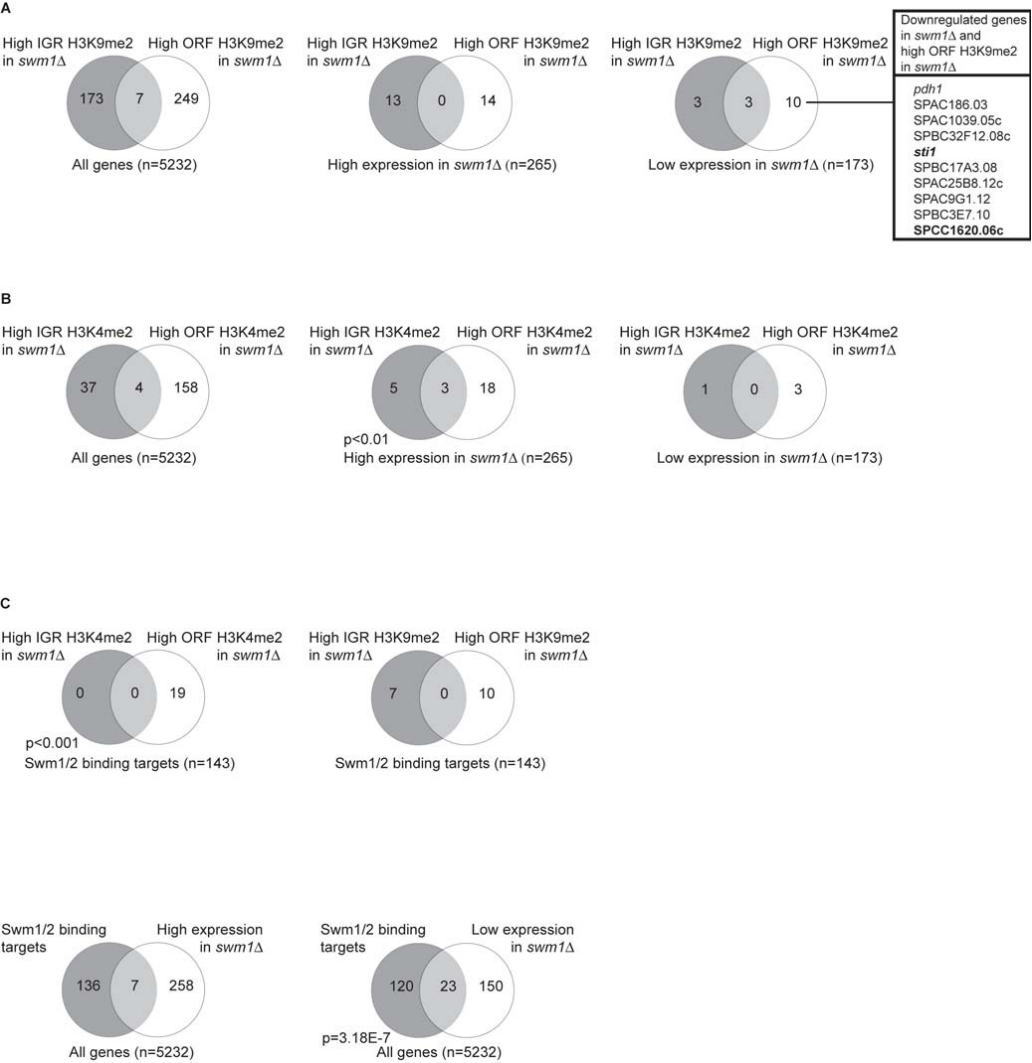
Genome wide analysis of histone methylation and gene expression in *swm1* cells, and binding of Swm1/2. A) A comparison of increased H3K9me2 and altered gene expression in *swm1* deletion cells. The Venn diagrams illustrate the degree of overlap between lists of IGR and ORF regions having high H3K9me2 in *swm1* deletion cells (using a cutoff value of 2.0 ), and a list of genes, which showed altered expression in *swm1*Δ (using a cutoff value of 1.5). Left: 8.2% of total *S. pombe* genes showed high IGR or ORF H3K9me2 levels in *swm1*Δ. Middle: The Venn diagram shows the fraction of genes up-regulated in *swm1*Δ having high IGR or ORF H3K9me2. Right: The Venn diagram shows the fraction of genes down-regulated in *swm1*Δ having high IGR or ORF H3K9me2. The inserted table shows a list of 10 genes down-regulated in *swm1* deletion cells, which also show high H3K9me2 ORF levels in *swm1*Δ (Swm1/2 binding targets are indicated in bold). B) A comparison of increased H3K4me2 and altered gene expression in *swm1* deletion cells. The Venn diagrams illustrate the degree of overlap between lists of IGR and ORF regions having high H3K4me2 in *swm1* deletion cells (using a cutoff value of 2.0 ), and a list of genes, which showed altered expression in *swm1* (using a cutoff value of 1.5). Left: 3.8% of total *S. pombe* genes showed high IGR or ORF H3K4me2 levels in *swm1*Δ. Middle: The Venn diagram shows the fraction of genes up-regulated in *swm1*Δ having high IGR or ORF H3K4me2. 9.8% of *swm1* up-regulated genes showed high IGR or ORF H3K4me2 levels, which is significantly more than expected from the genome average (CHI square, P<0.01; indicated). Right: The Venn diagram shows the fraction of genes down-regulated in *swm1*Δ having high IGR or ORF H3K4me2. C) Comparison of Swm1/2 binding targets (determined by Nicolas et al., (2006)), H3K4me2 and H3K9me2 levels, as well as gene expression changes in *swm1* deletion cells. Top: The Venn diagrams illustrate the degree of overlap between lists of IGR and ORF regions having high H3K4me2 and H3K9me2 levels (as indicated) in *swm1* deletion cells, and a list of Swm1/2-binding targets. 13.4% of Swm1/2 binding targets showed high IGR or ORF H3K4me2 levels, which is significantly more than expected from the genome average (CHI square, P>0.001; indicated). Bottom: The Venn diagrams illustrate the degree of overlap between the list of Swm1/2-binding targets and gene expression changes in *swm1* deletion cells. Bottom right: A significant proportion of s*wm1* down-regulated genes (hypergeometric P value indicated) were defined as Swm1/2-binding targets. (Note that our microarray contains only 143 of the 175 targets published by Nicolas et al., (2006).)

We emphasise, however, that Swm1 also has an activating role at 173 genes. The effect of *swm1* deletion on these genes appears to be direct. In hyper-geometric distribution tests, we found a statistically significant overlap when comparing the published genome-wide Swm1/2 localisation data [14] with the list of genes that are down-regulated in *swm1*Δ cells (P = 3.18×10-7), but not those that are up-regulated (compare Figure 4c bottom, left and right).

Further analysis, using hyper-geometric distribution tests, with a database of genes affected by histone modifications, showed that there is a very significant similarity of up-regulated genes in the *swm1*Δ strain to up-regulated genes in the *clr6-1* strain (see Table 1). *clr6-1* is a loss of function mutant of Clr6, a histone deacetylase (HDAC) and known transcriptional repressor, which is similar to HDAC1/2 in higher organisms [16]–[17]. Consistent with Swm1 acting in concert with Clr6, there is also a significant overlap of genes that are up-regulated in the *swm1*Δ strain with genes whose promoters lack acetylation in wild-type cells [18] (see Table 1).

**Table 1:** Hyper-geometric probability comparisons of genes either down- or up-regulated in *swm1* deletion cells.

HIGH Expression in *swm1*Δ vs wild-type, cut-off 1.5 (3 of 4), 265 genes	
P value	Similar list Name
1.25×10-33	stress up >2 fold
3.34×10-29	HIGH expression in *clr6-1 clr3* deletion (1.5 fold)
4.99×10-29	wild type meiosis up >2 fold
6.30×10-28	HIGH expression in *clr6-1* (1.5 fold)
3.82×10-11	LOW IGR WT H4K5Ac H3 Cter corr (1.5 fold)
8.42×10-09	LOW IGR WT H4K12Ac H3 Cter corr (1.5 fold)
1.00×10-06	LOW IGR WT H3K9Ac H3 Cter corr (1.5 fold)
5.48×10-06	IGR binding of Hrp1 (0.90 percentile)
8.92×10-06	HIGH expression in *clr3* deletion (1.5 fold)
1.33×10-05	IGR binding of Hrp3 (0.94 percentile)
7.14×10-05	LOW IGR WT H4K16Ac H3 Cter corr (1.5 fold)
0.000185	LOW IGR WT H3K14Ac H3 Cter corr (1.5 fold)
0.00022	Process-Carbohydrate Metabolism
0.0196	ORF binding Clr3-myc (0.86 percentile)

LOW Expression in *swm1*Δ vs wild-type, cut-off 1.5 (3 of 4), 173 genes	
P value	Similar list Name
2.03×10-07	HIGH expression in wild type
0.00014	Process-Amino Acid Metabolism
0.00418	GO: 0006531: Process: aspartate metabolism
0.0101	GO: 0006555: Process: methionine metabolism
0.0117	Process-Sulfur Metabolism
0.0496	Process-Transporters

Our analyses extend the observations of Nicolas et al., (2006) [14]. Thus, it is now clear that the *S. pombe* Swm1/2 complex has dual roles in gene regulation, up-regulating some 173 genes and down-regulating some 265 others. The correlation between Swm1/2 localisation and down-regulated genes in *swm1*Δ cells, suggests that the complex might have a more direct role in stimulating gene expression than it does in repression. Moreover, the imposition of a repressed state by Swm1/2 involves one of the HDACs – Clr6.

### The effects of Swm1 deletion on K4 and K9-methylation of histone H3

We next measured levels of H3K4 and H3K9 dimethylation using ChIP-chip experiments in both inter-genic (IGR) (including all pol II promoters) and open-reading frame (ORF) regions in the *swm1*Δ strain [see Ref. 18 for details of the microarray platform]. (Our studies focussed on the levels of dimethylation, as opposed to monomethylation, because of the availability of suitable antibodies. In addition, levels of trimethylation are known to be low in *S. pombe*.) In agreement with our finding that the fission yeast Swm complex demethylates H3K9 (Figure 2), deletion of *swm1* resulted in increased levels of H3K9me2 in a large part of the genome – 8.2% of genes showed increased H3K9me2 in either the ORF or IGR regions. H3K4me2 levels, however, were also increased in a smaller part of the genome (3.8%). Interestingly, there appears to be a bias towards increased levels of H3K4me2 in ORF regions (as compared to IGR regions), whereas H3K9me2 levels were increased in both the ORF and IGR regions (compare Figures 4a, 4b). (See Table S3 in the Supplementary information for lists of the IGR and ORF regions with high levels of H3K4me2 and H3K9me2 in the *swm1*Δ strain.) (Note that the *S. pombe* genome is composed of roughly equal amounts of IGR and ORF regions.)

Globally, overall levels of H3K9me2 were not significantly changed in genes whose expression is either up- or down-regulated in the absence of Swm1 (Figure 4a). However, on closer examination of the genes that are activated by Swm1, a few have increased H3K9me2 levels in the absence of Swm1. Within this group of 10 genes, *sti1*+ and SPCC1620.06c were previously identified as Swm1/2 binding targets [14]. This data suggests that in wild-type cells Swm1 may have an activating function at some genes, which involves demethylation of H3K9me2 in ORF regions, consistent with the general role of H3K9me2 in gene repression and silencing in *S. pombe* [19]–[21]. However, due to the small number of genes involved, this does not appear to be the major role of the Swm1/2 complex.

In genes that are repressed by Swm1, and therefore up-regulated in the *swm1*Δ strain, levels of H3K4me2 were seen significantly more frequently (CHI square, P<0.01). The bias towards increased levels of H3K4me2 in ORF regions (as compared to the IGR regions) was again apparent (Figure 4b). However, no difference in H3K4me2 levels was observed in genes that are activated by Swm1, and therefore repressed in the *swm1*Δ strain.

The bias towards increased H3K4me2 levels in ORF regions of genes that are up-regulated in *swm1*Δ cells, when taken together with the lack of *in-vitro* H3K4 demethylase activity of the Swm1/2 complex (see above), suggests that the increased H3K4me2 levels results from either increased H3K4 methylation via Set1, or the incorporation of H3K4 methylated histones during transcription.

### Correlation of H3K9me2 or H3K4me2 levels with other post-translational modifications

The changes in H3K9me2 or H3K4me2 levels observed at genes in the *swm1*Δ cells may correlate with other post-translational modifications associated with the same genes. To test this we compared the lists of IGR and ORF regions showing either high H3K9me2 or H3K4me2 in *swm1*Δ cells with our database of genes affected by histone modifications using hyper-geometric distribution tests.

The regions which show increased H3K4me2 in the *swm1*Δ cells were very significantly similar to regions where histone acetylation is low in wild-type cells. They were also significantly similar to regions with increased acetylation in the *clr6-1* mutant [18] (Table 2). These findings are consistent with the observation that there is a very significant similarity of up-regulated genes in the *swm1*Δ strain to up-regulated genes in the *clr6-1* strain (see above and Table 1). The functional link to Clr6 is also interesting given that LSD1 (a human homologue of Swm1) physically and functionally interacts with HDAC1/2 [7]–[10]. Moreover, the regions associated with increased H3K4me2 in *swm1*Δ cells tend to be the 3′ regions of longer genes (>1000 bp), which in wild type cells normally have low levels of both histone acetylation and H3K4me2, and which are hyperacetylated in *clr6-1* cells [22]. These findings again suggest that Swm1 and Clr6 may collaborate to maintain repressive chromatin in both IGR and ORF regions, to influence both transcriptional initiation and elongation. Furthermore, the affected IGR regions in the *swm1*Δ cells also overlap significantly with Clr3 and Sir2 HDAC binding data (Table 2).

**Table 2:** Hyper-geometric probability comparisons of IGR and ORF lists with high H3K4me2 in *swm1* deletion cells.

HIGH IGR H3K4me2 in *swm1*Δ, 41 genes	
P value	Similar list Name
1,64×10-19	High IGR *clr6-1* H4K5ac H3 cter corr (2 fold )
2,33×10-18	LOW IGR WT H3K14Ac H3 Cter corr (1.5 fold)
2,82×10-16	High IGR *clr6-1* H4K12ac H3 cter corr (2 fold )
1,82×10-15	High IGR *clr6-1* H3K14ac H3 cter corr (2 fold)
5,59×10-15	LOW IGR WT H4K5Ac H3 Cter corr (1.5 fold)
7,23×10-15	LOW IGR WT H3K9Ac H3 Cter corr (1.5 fold)
7,31×10-13	LOW IGR WT H4K12Ac H3 Cter corr (1.5 fold)
7,64×10-13	High IGR *clr6-1* H4K16ac H3 cter corr (2 fold )
6,45×10-12	LOW IGR WT H3K4me2 H3 cter corr (1.5 fold)
2,11×10-9	LOW IGR WT H4K16Ac H3 Cter corr (1.5 fold))
6,05×10-8	High IGR *clr6-1* H3K9ac H3 cter corr (2 fold )
9,01×10-6	Hrp1 IGR binding (0.9 percentile)
0,000564	Hrp3 IGR binding (0.94 percentile)
0,00058	HIGH expression in wild type meiosis (2 fold)
0,00303	IGR Binding Sir2-myc (0.91 percentile)
0,0497	IGR binding Clr3-myc (0.86 percentile)

HIGH ORF H3K4me2 in *swm1*Δ, 162 genes	
P value	Similar list Name
2,65×10-28	LOW ORF WT H3K14Ac H3 Cter corr (1.5 fold)
2,4×10-23	LOW ORF WT H4K5Ac H3 Cter corr (1.5 fold)
3,07×10-20	LOW ORF WT H4K16Ac H3 Cter corr (1.5 fold)
3,65×10-20	Gene length (>1000)
6,0××10-18	LOW ORF WT H4K12Ac H3 Cter corr (1.5 fold)
1,1×10-9	Gene length (>2000)
4,31×10-6	Hrp3 ORF binding (0.92 percentile)
6,66×10-6	LOW ORF H3K4Met H3 Cter corr (1.5 fold)
2,99×10-5	LOW IGR WT H4K12Ac H3 Cter corr (1.5 fold)
5,46×10-5	LOW ORF WT H3K9Ac H3 Cter corr (1.5 fold)
0,000123	Swm1/2 binding targets
0,000535	High ORF *clr6-1* H4K12Ac H3 cter corr (2 fold)
0,0249	High ORF *clr6-1* H4K16Ac H3 cter corr (2 fold)
0,0324	High ORF *clr6-1* H4K5Ac H3 cter corr (2 fold)
0,0455	High ORF *clr6-1* H3K14Ac H3 cter corr (2 fold)

HIGH IGR or ORF H3K4me2 in *swm1*Δ, combined list 199 genes	
P value	Similar list Name
0,0032	Swm1/2 binding targets
0,0133	HIGH Expression in *swm1*Δ (1.5 fold)

No similar gene lists were found for ‘HIGH IGR H3K9me2 in *swm1*Δ’ and ‘HIGH ORF H3K9me2 in *swm1*Δ’

The lists of IGR regions which show increased levels of H3K4me2 in the *swm1*Δ strain also overlapped significantly with lists of genes representing IGR binding of the Hrp1 chromatin remodelling factor and its closely related (64% identical) paralogue Hrp3 (P = 9.01×10-6 and 5.64×10-4, respectively; see Table 2). Moreover, the lists of up-regulated genes in the *swm1*Δ strain showed significant similarity to lists of IGR regions bound by Hrp1 and Hrp3 (P = 5.48×10-6 and 1.33×10-5, respectively; See Table 1).

### Possible targeting of the Swm complex via Clr6?

Given the finding that loss of function of either the Swm1 histone demethylase or Clr6 HDAC, results in up-regulation of genes and altered levels of histone modifications in the same regions of the genome, and the knowledge that human LSD1 physically and functionally interacts with HDAC1/2 [7]–[10], we wondered whether there was a physical interaction between the *S. pombe* Swm1 and Clr6 complexes? Because we had not identified any of the Clr6 complex components in TAP-tagging/MS studies of Swm1 and Swm2, we also TAP-tagged components of the Clr6 complex, to see if we could identify any of the Swm partner proteins. These studies, however, only identified the same proteins found in previous work [23], namely: Clr6, Alp13, Prw1 and Pst2 (data not shown). In preliminary experiments we have found that Hrp1 and Hrp3 co-purify in affinity purification experiments (Walfridsson, Khorosjutina, Gustafsson, Ekwall *et. al.* manuscript in preparation). Our results, therefore, indicate that there is a conserved functional interaction between the Swm1 histone demethylase and the Clr6 histone deacetylase that is also found in mammalian cells. They also provide further support for a model in which deacetylation of nucleosomes sets the stage for demethylation [12]. In *S. pombe*, however, despite the suggested functional association, and unlike the situation in higher organisms, any interaction between the two complexes appears to be of a more transient character.

### Possible targeting of the Swm complex via Hrp1/3?

Previously, Nicolas et al (2006) [14] showed that the Swm1/2 complex physically interacts with Hrp1. The putative functional links to the Hrp1 and Hrp3 chromatin remodelling factors that we identify here suggest that this interaction may provide a mechanism for targeting of the Swm demethylase. The double chromo domains of human CHD1, but not those of *S. cerevisiae* Chd1p, have been shown to bind directly to methylated H3K4 [24]–[25]. Although the *S. pombe* Hrp1, does not have all the consensus residues required for methyl-lysine binding, Hrp3 does (data not shown). Because Hrp1 and Hrp3 co-purify we speculate that Hrp1/Swm complex interactions might target some Swm complexes to perform demethylation through the interaction with Hrp3.

### Concluding remarks

In conclusion, the data presented here demonstrate that in fission yeast the SWIRM/PAO domain proteins Swm1 and Swm2 are associated in complexes that can remove methyl groups from lysine 9 methylated histone H3. Interestingly, recognition of the H3K9 substrate appears to require an as yet unidentified modification. *In vivo*, loss of Swm1 increases the global levels of H3K9me2 and H3K4me2, and it results in a significant accumulation of H3K4me2 at genes that are up-regulated in the *swm1*Δ strain. The bias towards increased H3K4me2 levels in ORF regions of genes that are up-regulated (as compared to IGR regions), suggests that this increase results from either increased methylation by Set1, or incorporation of H3K4 methylated histones, during transcription.

An alternative explanation for the increased levels of H3K4me2 is that Hrp1/3 might act as coregulators, and influence the specificity of the Swm1 complex. In a similar manner to that of human LSD1, which when complexed with the androgen receptor switches from a H3K4 to a H3K9 demethylase [13], it is possible that K4 demethylation is favored over K9 in the functional context of the chromatin remodelling factors Hrpl and Hrp3. However, the bias to increased levels of H3K4me2 in ORF regions, suggests (as discussed above) that the increased levels at these genes results in some way from increased transcription.

Aside from at a few genes, where in the *swm1* deletion increased H3K9me2 levels are correlated with reduced levels of gene expression, the functional role of the Swm1/2 H3K9 demethylase activity is not yet clear. Our results, however, highlight complex interactions between histone demethylase, histone deacetylase and chromatin remodelling activities in the regulation of gene expression. The *in vivo* data indicate that Swm1 acts in concert with the HDAC Clr6 and the chromatin remodeller Hrp1 to repress gene expression, but further work is necessary to uncover the nature of these functional interactions.

Supplementary information is available on line.

## Methods

### Reagents

IgG Sepharose was from Amersham, Ni-magnabeads were from Promega and the Dynabeads M280 were from Dynal Biotech. Rabbit anti-mouse immunoglobulin protein used for coating the beads was from DAKO, A/S Denmark. Calf thymus bulk histones and histone H3 were purchased from Roche. Chicken polynucleosomes were purchased from Abcam and recombinant Xenopus laevis H3 was from Upstate.

### Construction of strains

Strains expressing TAP-tagged Swm1 and Swm2, and the *swm1* knockout strain, were constructed using previously described protocols [26]–[28]. In brief, the TAP-tagging constructs for homologous recombination were made using fusion PCR of 500 base pair genomic DNA fragments and the C-terminal TAP-tagging cassette. The resulting DNA fragment was transfected into S. pombe strain 501 *(leu1-32; ura4-D18; ade6-704;h^−^).* Homologous recombination was confirmed by PCR with primers inside and outside the incorporated TAP-tag. Expression of the construct and fusion protein solubility was checked by Western blots with anti-protein A antibodies. Strain viability was compared to that of the wild type strain in normal growth and under stress conditions (37°C). The *swm1*Δ knockout strain was constructed by homologous integration of Clonat, replacing the ORF. The integration was confirmed by PCR using primers 5′ and 3′ to the recombined region together with primers internal to the Clonat gene.

### Cells growth and affinity purification of the complexes

The published TAP-purification procedure [29] was followed with minor changes. The wild type and TAP-tagged strain cells were harvested and washed in ice-cold water, resuspended in 20 mM HEPES pH 8.0, 150 mM NaCl, 0.1% Tween 20 and broken in liquid nitrogen using a SPEX CertiPrep 6850 Freezer Mill. The soluble fraction was bound to either IgG coated Sepharose or IgG coated Dynabeads, washed extensively, cut with His-tagged TEV protease and eluted from the beads. Excess IgG and TEV were removed by incubation with protein A and Ni-agarose beads. The eluate was bound to calmodulin-binding beads and eluted with EGTA containing buffer. To avoid loss of protein the complex was not further purified by SDS-PAGE. Instead, the eluted complex was directly digested with trypsin and the resulting peptides identified by mass- spectrometry.

### Purification of hLSD1

Human LSD1 (residues 72-852) was subcloned into the pET30 vector (Novagen). Protein expression was carried out in the *E. coli* Rossetta 2 strain (Novagen). To induce expression 0.5 mM IPTG was added to cultures at OD_600_ of 0.6 and incubated at 25°C for 4 hours. Harvested cells were disrupted in lysis buffer (40 mM Tris pH 8.0, NaCl 300 mM, 0.5% NP40, protease inhibitors (Sigma)) using an Emulsiflex-05 (Avestin) at up to 8000 psi. hLSD1 was purified from the clarified lysate with Ni-NTA agarose (Qiagen) and further purified by anion exchange chromatography (MonoQ column (Pharmacia Biotech)) and gel filtration (Superdex 200 (Pharmacia Biotech)) using standard protocols.

### Purification of GST-Swm1

Full length GST-Swm1 was subcloned into pGEX-2T. Protein expression was carried out in the *E.coli* Rossetta strain. To induce expression 0.5 mM IPTG was added to cultures at an OD_600_ of 0.5 and incubated at 15°C for 16 hours. Harvested cells were disrupted in lysis buffer (25 mM Tris-HCL pH 8.0, 150 mM NaCl, 0.1% Triton-X-100, protease inhibitors (Roche)) by sonication. Glutathione sepharose resin (Pharmacia) was added to the clarified lysate and incubated with rotation for 2 hrs at 4°C. The resin was then extensively washed with lysis buffer without Triton-X-100. Bounds proteins were finally eluted in 50 mM Tris pH 8.0, 10 mM glutathione.

### In-vitro demethylase assays

^3^H-labelled methyl histone substrates were prepared using the following histone methyltransferases (HMT): GST-Set7, MBP-Clr4 and GST-Set2 as previously described [15]. Briefly, 100 ul labelling reactions were carried out in methylase buffer (50 mM Tris pH 8.0, 1 mM DTT, 10% glycerol and 10 µM ZnCl_2_) containing 1–5 ug HMTs, 5 µl S-adenosyl-[^3^H]methyl-methionine (70 Ci/mmol, NEN) and substrate (50 µg calf thymus bulk histones, 10 µg chicken poly-nucleosomes, 10 µg calf thymus histone H3 or 10 µg recombinant Xenopus laevis H3). After a 5 minute incubation at 37°C, reactions were immediately dialysed into demethylase buffer (50 mM Tris pH 8.8, 0.5 mM DTT and 5% glycerol) at 4°C.

For demethylase assays labelled histone substrates containing 5,000–50,000 cpm were incubated with 25 ul of either TAP-tagged Swm complex, mock purifications from wild-type cells, or 5 ug hLSD1 or GST-Swm1 in a final volume of 100 ul with demethylase buffer at 37°C for 1 hr. The Nash method was then used to detect the formation of ^3^H-labelled formaldehyde [30]. After precipitation with 10% TCA an equal volume of Nash reagent (3.89 M ammonium acetate, 0.1 M acetic acid and 0.2% 2,4-pentanedione) was added to the supernatant and incubated at 37°C for 50 minutes, followed by extraction with an equal volume of 1-pentanol. The extracted supernatant was then subjected to scintillation counting.

### Genome wide analysis

The microarray analysis in this study was performed essentially as outlined in [18]. cDNA expression profiling was carried out according to Xue et al., (2004) [31]. We used *S. pombe* ORF and combined IGR+ORF spotted microarrays from Eurogentec custom DNA microarray services (Belgium). For histone methylation maps, ChIP-chip experiments were carried according to Robyr and Grunstein (2003) [32]. Antibodies recognising specific methylation marks were employed: H3K9me2 and H3K4me2 (a kind gift from Prof. David Allis). For Hrp1 and Hrp3 binding studies we used the ChIP-chip procedure described by Kurdistani et al. (2002) [33]. For expression profiling and analysis of histone methylation levels, two microarray experiments were performed using two independent ChIP samples and including Cy3/Cy5 dye swops. Because each Eurogentec microarray yields two data points we thus measured four data points for each experiment. Expression profiling and histone methylation in mutant *vs*. wild-type data sets were normalized using the GeneSpring software and Lowess (per spot, per chip) intensity-dependent normalization, which corrects for nonlinear rates of dye incorporation. Cut off values of 1.5 (for gene expression) and 2.0 (for histone methylation levels) in at least 3 out of 4 data points were used to generate the ‘high’ and ‘low’ gene lists. For Hrp1 and Hrp3-binding experiments three microarrays were used, yielding six data points. Here we first used the GeneSpring software for a ‘per chip’ normalization and then employed a statistically determined cut-off for binding according to the median percentile ranking method [34]. Similar gene lists were identified using the automatic hyper-geometric distribution tests in the Gene List inspector function of Gene Spring. The hyper-geometric distribution test calculates the probability of overlap corresponding to *k* or more IGR or ORF fragments between an IGR or ORF list of *n* fragments compared against another gene list of *m* fragments when randomly sampled from a universe of *u* genes:
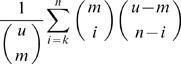
The significantly overlapping gene lists were illustrated using Venn diagrams.

## Supporting Information

Table S1 a) Identification of peptides in the Swm complex purified using TAP-tagged Swm1 (SPBC146.09C) b) Identification of peptides in the Swm complex purified using TAP-tagged Swm2 (SPAC23E2.02)
(0.16 MB DOC)

Table S2 A list of genes either down- or up-regulated in swm1 deletion cells
(0.17 MB DOC)

Table S3 Genes that have either increased levels of H3K4me2 or H3K9me2 in swm1 deletion cells
(0.31 MB DOC)
