# Supplementary material for: Genome-Wide Studies of Histone Demethylation Catalysed by the Fission Yeast Homologues of Mammalian LSD1
Source: PLoS One. 2007 Apr 18;2(4):e386. doi: 10.1371/journal.pone.0000386 (PMC1849891; doi:10.1371/journal.pone.0000386)
Supplement: Table S1 — a) Identification of peptides in the Swm complex purified using TAP-tagged Swm1 (SPBC146.09C) b) Identification of peptides in the Swm complex purified using TAP-tagged Swm2 (SPAC23E2.02) (0.16 MB DOC) [file pone.0000386.s001.doc]

**Table S1**

**a)** Identification of peptides in the Swm complex purified using TAP-tagged Swm1 (SPBC146.09C)

(For each protein the peptides identified are shown.)

| Protein | Peptide sequence | Position |
| --- | --- | --- |
| Swp1 | KIQRPVAYNPNATALK | 166-181 |
|  | AKQPLETGMTAQDLGLSESDKK | 248-269 |
|  | SYPSLPIYNPR | 290-300 |
|  | ELLGEIR | 303-309 |
|  | HQLLVSSER | 310-318 |
|  | QQISLQER | 319-326 |
|  | QDEAPSDEPAPVPYTASYVANSGTLYDYPTLIR | 331-363 |
|  | SGSIYQIATVEEYPHLQPSLLPTFQR | 411-436 |
| Swp2 | SEQAPFTEDASSSNYAHHR | 111-129 |
|  | SWPASLAIER | 164-173 |
|  | DNTADALFSTEDGREEQFNLEGVK | 174-197 |
|  | KVHPRNHFDPLVK | 203-215 |
|  | RPVVALAMK | 226-234 |
|  | QAPLVTGTTAR | 291-301 |
|  | GSSYELNR | 478-485 |
| Swm2 | VLLQWSSQSSSHTIPSAGASIPTSSLK | 362-388 |
|  | SFFEHAAEAAR | 389-399 |
|  | KCNLDPRALESFEQHMLSDR | 400-419 |
|  | LHDPVVLFHYFQIR | 420-433 |
|  | NSICWLWIK | 434-442 |
|  | QLTGLFSQYSSSFLSK | 524-539 |
|  | ALPVSHTSATQINHHTSNSNSISSNSTSLNPK | 563-594 |
|  | VLDMVEVKKLNILWEK | 650-665 |
|  | SLSLNASHEFSFK | 705-717 |
|  | FLIWCFK | 740-746 |
|  | VAELDDTLYPLNTVDTDFSK | 747-766 |
|  | NGVYDGLNSYPNFANDK | 969-985 |
|  | QSQLSYNLGR | 994-1003 |
|  | LHIAGDYIFSCVGCR | 1004-1018 |
|  | ELDQLFR | 1055-1061 |
|  | VNNFDPNAEAQR | 1065-1076 |
|  | HLSYQAR | 1077-1083 |
|  | MQWHVCR | 1128-1134 |
|  | KPWDEIAAQR | 1174-1184 |
|  | CQDEPIPDDEARLFMQAQR | 1216-1234 |
| Swm1 | GRPALNTSNSLER | 111-123 |
|  | SSIPAIPR | 139-146 |
|  | NGICYLWHR | 193-201 |
|  | NPTLYVSFNEALGIVREKK | 202-220 |
|  | KAFPLASLAFEFLSR | 220-234 |
|  | QLTNLFAQYEQDFLSR | 275-290 |
|  | IVIYEASER | 297-305 |
|  | EFILHDIENGR | 377-387 |
|  | IDTEHVQR | 388-395 |
|  | IFEWFK | 475-480 |
|  | LNTHLITFEPPLEEK | 575-589 |
|  | FYEHPTLSVFVK | 640-651 |
|  | SEAINPIR | 679-686 |
|  | RISYHTEYLR | 803-812 |
|  | ISYHTEYLRLRQK | 804-816 |
|  | QPWVDEINAR | 900-909 |
|  | RANYSTR | 910-916 |
|  | LEEYQR | 917-922 |
|  | WINSYNVR | 923-930 |
|  | LLAEQEDEHLHPEK | 956-969 |

**b)** Identification of peptides in the Swm complex purified using TAP-tagged Swm2 (SPAC23E2.02)

(For each protein the peptides identified are shown.)

| **Protein** | **Peptide sequence** | **Position** |
| --- | --- | --- |
| Swp1 | NGRIRSSR | 121-128 |
|  | REDDNR | 129-134 |
|  | IQRPVAYNPNATALK | 166-181 |
|  | SYPSLPIYNPR | 290-300 |
|  | ELLGEIR | 303-309 |
|  | QQISLQER | 319-326 |
|  | QDEAPSDEPAPVPYTASYVANSGTLYDYPTLIR | 331-363 |
| Swm1 | SSIPAIPR | 139-146 |
|  | NPTLYVSFNEALGIVR | 202-217 |
|  | KAFPLASLAFEFLSR | 220-234 |
|  | QLTNLFAQYEQDFLSR | 275-290 |
|  | IVIYEASER | 297-305 |
|  | EFILHDIENGR | 377-387 |
|  | IDTEHVQR | 388-395 |
|  | LNTHLITFEPPLEEK | 575-589 |
|  | FYEHPTLSVFVK | 640-651 |
|  | SEAINPIR | 679-686 |
|  | RISYHTEYLR | 803-812 |
|  | ISYHTEYLRLRQK | 804-816 |
|  | QPWVDEINAR | 900-909 |
|  | RANYSTR | 910-916 |
|  | LEEYQR | 917-922 |
|  | LLAEQEDEHLHPEK | 956-969 |
| Swm2 | TIGLPPTVGSSFPQQK | 179-194 |
|  | SSTYENFFDANSPSSQQFPSTYPSR | 195-219 |
|  | DQVSHGVTPSTFR | 270-282 |
|  | NHESFMPTQLVSATELSK | 283-300 |
|  | VLLQWSSQSSSHTIPSAGASIPTSSLK | 362-388 |
|  | SFFEHAAEAAR | 389-399 |
|  | KCNLDPRALESFEQHMLSDR | 400-419 |
|  | LHDPVVLFHYFQIR | 420-433 |
|  | NSICWLWIK | 434-442 |
|  | VEAQGVCVDR | 451-460 |
|  | TIAVVGAGLTGLICAR | 508-523 |
|  | QLTGLFSQYSSSFLSK | 524-539 |
|  | ALPVSHTSATQINHHTSNSNSISSNSTSLNPK | 563-594 |
|  | QLGIEVTEMTGSDLVYDETDTK | 628-649 |
|  | SLSLNASHEFSFK | 705-717 |
|  | TMLILIDEVSSYAK | 719-732 |
|  | VAELDDTLYPLNTVDTDFSK | 747-766 |
|  | GLSQLPWALQSYPSPLNIHYEK | 777-798 |
|  | YAAAFWR | 867-873 |
|  | NGVYDGLNSYPNFANDK | 969-985 |
|  | QSQLSYNLGR | 994-1003 |
|  | ELDQLFR | 1055-1061 |
|  | VNNFDPNAEAQR | 1065-1076 |
|  | HLSYQAR | 1077-1083 |
|  | ATINETR | 1152-1158 |
|  | KPWDEIAAQR | 1174-1184 |
|  | EASDEEYHDDGSSDSGYNGTR | 1252-1272 |
