# Supplementary material for: Genome-Wide Studies of Histone Demethylation Catalysed by the Fission Yeast Homologues of Mammalian LSD1
Source: PLoS One. 2007 Apr 18;2(4):e386. doi: 10.1371/journal.pone.0000386 (PMC1849891; doi:10.1371/journal.pone.0000386)
Supplement: Table S2 — A list of genes either down- or up-regulated in swm1 deletion cells (0.17 MB DOC) [file pone.0000386.s002.doc]

**Table S2 –** A list of genes either down- or up-regulated in *swm1* deletion cells

(We used a cutoff value of 1.5 to define genes whose expression was affected in at least 3 out of 4 experiments by the *swm1* deletion – see main text for further details).

| **Low expression in swm1 vs wt cut 1,5 (3 of 4) 173 genes** | **High expression in swm1 vs wt cut 1,5 (3 of 4) 265 genes** |
| --- | --- |
|  |  |
| SPAC1039.01 | SPBC1271.15c |
| SPAC1039.02 | SPBC1271.03c |
| SPAC1039.04 | SPBC1198.01 |
| SPAC1039.05c | SPBC16C6.09 |
| SPAC1039.06 | SPBC16G5.02c |
| SPAC1039.08 | SPBC16D10.08c |
| SPAC11D3.13 | SPBC119.05c |
| SPAC11D3.18c | SPBC106.13 |
| SPAC1250.04c | SPAPB24D3.07c |
| SPAC1348.12 | SPBC11C11.06c |
| SPAC139.02c | SPBC1271.05c |
| SPAC14C4.12c | SPBC119.03 |
| SPAC16.03c | SPBC27.04 |
| SPAC1782.06c | SPBC21B10.07 |
| SPAC186.03 | SPBC215.16c |
| SPAC186.08c | SPBC29A10.08 |
| SPAC19G12.05 | SPBC29A10.06c |
| SPAC19G12.13c | SPBC27.05 |
| SPAC1A6.10 | SPBC18H10.05 |
| SPAC1A6.11 | SPBC1683.01 |
| SPAC1B2.03c | SPBC16E9.16c |
| SPAC1B3.01c | SPBC1E8.05 |
| SPAC1B3.13 | SPBC19C7.04c |
| SPAC1B3.16c | SPBC17G9.06c |
| SPAC222.08c | SPAC2G11.13 |
| SPAC22A12.10 | SPAC2F3.05c |
| SPAC22A12.12c | SPAC29A4.01c |
| SPAC22A12.16 | SPAC3G9.11c |
| SPAC22G7.05 | SPAC3C7.05c |
| SPAC23C11.12 | SPAC30.05 |
| SPAC23C4.06c | SPAC24B11.12c |
| SPAC23G3.05c | SPAC23H3.15c |
| SPAC23H3.12c | SPAC23G3.13c |
| SPAC25B8.12c | SPAC27F1.05c |
| SPAC25B8.15c | SPAC27D7.09c |
| SPAC29B12.10c | SPAC27D7.10c |
| SPAC2E1P3.05c | SPAC806.09c |
| SPAC2F3.04c | SPAC750.02c |
| SPAC30D11.02c | SPAC6F6.01 |
| SPAC3A12.04c | SPAPJ691.02 |
| SPAC4C5.01 | SPAPB2B4.04c |
| SPAC4C5.05c | SPAC869.02c |
| SPAC56E4.03 | SPAC5D6.04 |
| SPAC56F8.09 | SPAC4F10.07c |
| SPAC5H10.06c | SPAC4D7.02c |
| SPAC630.08c | SPAC6B12.07c |
| SPAC631.03 | SPAC688.04c |
| SPAC869.08 | SPAC637.03 |
| SPAC8F11.09c | SPCC4G3.13c |
| SPAC922.07c | SPCC4G3.06c |
| SPAC9G1.12 | SPCC4G3.07c |
| SPACUNK4.19 | SPCC576.04 |
| SPAP32A8.03c | SPCC736.13 |
| SPAP8A3.11c | SPCC584.15c |
| SPAPJ760.03c | SPCC417.13 |
| SPBC106.17c | SPCC4B3.01 |
| SPBC1105.01 | SPCC338.18 |
| SPBC11G11.03 | SPCC4G3.03 |
| SPBC1271.07c | SPCC4B3.13 |
| SPBC1271.10c | SPCC4G3.09c |
| SPBC1271.11 | wtf10-pseudo |
| SPBC1289.16c | tpp1 |
| SPBC12C2.14c | thi3 |
| SPBC146.09c | wtf6 |
| SPBC1683.05 | wtf3-pseudo |
| SPBC16C6.12c | wtf13 |
| SPBC16E9.05 | ste4 |
| SPBC1703.05 | SPCC757.03c |
| SPBC1709.02c | SPCC645.06c |
| SPBC1709.03 | tea2 |
| SPBC1711.04 | SPCC970.11c |
| SPBC1711.07 | ste7 |
| SPBC1734.01c | SPBC800.14c |
| SPBC1778.07 | SPBC713.11c |
| SPBC17A3.04c | SPBC56F2.09c |
| SPBC17A3.08 | SPCC1322.04 |
| SPBC19F5.04 | SPBC83.10 |
| SPBC21.08c | SPBC83.13 |
| SPBC211.05 | SPBC3B8.07c |
| SPBC23E6.05 | SPBC365.12c |
| SPBC29A3.01 | SPBC32F12.10 |
| SPBC2G2.12 | SPBC428.10 |
| SPBC30B4.07c | SPBC3F6.06c |
| SPBC32F12.08c | SPBC3B9.16c |
| SPBC342.02 | SPCC1393.12 |
| SPBC359.02 | SPCC1322.10 |
| SPBC359.03c | SPCC1281.09 |
| SPBC359.05 | SPCC338.12 |
| SPBC365.16 | SPCC306.08c |
| SPBC3D6.08c | SPCC306.11 |
| SPBC3D6.12 | SPBPB2B2.13 |
| SPBC3E7.10 | SPBP4G3.03 |
| SPBC3H7.07c | SPBP23A10.12 |
| SPBC409.13 | SPCC1183.09c |
| SPBC428.05c | SPBPB2B2.18 |
| SPBC428.11 | SPBPB2B2.19c |
| SPBC4F6.07c | rho2 |
| SPBC4F6.13c | rgs1 |
| SPBC530.07c | rec10 |
| SPBC646.06c | rpn2 |
| SPBC725.15 | rip1 |
| SPBC776.03 | pmd1 |
| SPBC776.14 | pi004 |
| SPBC839.16 | php3 |
| SPBC8D2.18c | rhp4a |
| SPBP4H10.15 | psk1 |
| SPBPB10D8.04c | SPAC144.10c |
| SPBPB10D8.05c | SPAC13C5.05c |
| SPBPB10D8.07c | SPAC13C5.04 |
| SPBPB7E8.01 | SPAC15E1.10 |
| SPCC1235.11 | SPAC15E1.02c |
| SPCC1235.12c | SPAC1006.01 |
| SPCC1620.06c | sod2 |
| SPCC1672.03c | pof2 |
| SPCC1672.07 | SPAC14C4.07 |
| SPCC1682.09c | SPAC1348.11 |
| SPCC17D1.06 | SPAC1348.03 |
| SPCC1827.06c | cta1 |
| SPCC2H8.04 | csx1 |
| SPCC569.07 | coq5 |
| SPCC663.05c | gos1 |
| SPCC736.12c | exg1 |
| SPCC777.09c | bgl2 |
| SPCC965.14c | atp7 |
| SPCP1E11.10 | atf1 |
| SPCPB1C11.03 | dak2 |
| ade2 | cmk1 |
| ade5 | matmc |
| apl2 | itr1 |
| ask2 | isp6 |
| ccr1 | pho1 |
| ceg1 | itr2 |
| clr4 | hsp16 |
| csn1 | hsp9 |
| cut6 | cki2 |
| cyp3 | mde6 |
| cyp9 | idh1 |
| dbp2 | fbp1 |
| dfr1 | SPAC22H10.13 |
| dld1 | SPAC18G6.01c |
| eng1 | SPAC20G4.03c |
| fio1 | SPAC16E8.03 |
| fur4 | SPAC1786.02 |
| gln1 | SPAC17G8.11c |
| gpd2 | SPAC22F8.05 |
| ilv1 | SPAC18B11.04 |
| klp3 | SPAC212.11 |
| mde3 | SPAC1B3.06c |
| meu13 | SPAC23C11.06c |
| nic1 | SPAC1F7.03 |
| pdh1 | SPBPB2B2.16c |
| pep1 | SPAC212.02 |
| ppa | SPCC338.06c |
| prp1 | SPCC417.05c |
| pss1 | SPAC2F3.08 |
| rad2 | grx1 |
| rec7 | gpx1 |
| rpl16c | SPCC4F11.05 |
| rst2 | gpd1 |
| sec23b | SPAC212.05c |
| sfc3 | SPBPB2B2.12c |
| sid1 | SPCC14G10.05 |
| sou1 | SPBPB7E8.02 |
| spf31 | SPCC1620.09c |
| sti1 | leu2 |
| tcg1 | SPAC3A11.10c |
| tif11 | mam2 |
| ura1 | SPCC16C4.03 |
| uvi22 | SPAC31A2.14 |
| vps13b | SPAC2G11.14 |
| wtf-pseudo | idh2 |
| ydc2 | SPAC20H4.11c |
| ypt2 | map1 |
|  | SPCC320.02c |
|  | fip1 |
|  | Tf2-11 |
|  | vps1 |
|  | vps27 |
|  | cut2 |
|  | tf2-10 |
|  | tf2-6 |
|  | aur1 |
|  | alp16 |
|  | wtf2-pseudo |
|  | SPAC23A1.14c |
|  | matmc-x2 |
|  | atp16 |
|  | wtf5 |
|  | wtf7 |
|  | GST |
|  | SPCC622.02 |
|  | SPCC622.11 |
|  | SPCC569.02c |
|  | SPAC22A12.17c |
|  | ecm2 |
|  | dak1 |
|  | SPAC222.11 |
|  | SPAC25B8.03 |
|  | SPAC24H6.02c |
|  | SPCC794.04c |
|  | SPCPB16A4.07c |
|  | SPAC26F1.04c |
|  | SPCC777.03c |
|  | cox4 |
|  | SPAC1348.02 |
|  | SPAC12B10.10 |
|  | SPAC11H11.05c |
|  | SPBC1709.12 |
|  | SPAC1348.05 |
|  | SPBC15D4.11c |
|  | SPBC1685.08 |
|  | SPAC750.04c |
|  | SPBC24C6.09c |
|  | SPAC17G6.19c |
|  | SPBC577.03c |
|  | SPAC664.15 |
|  | SPAC6G10.03c |
|  | SPAC1002.12c |
|  | SPBC19C2.09 |
|  | SPAC1635.01 |
|  | SPAC1687.07 |
|  | SPAC15A10.09c |
|  | SPAC824.07 |
|  | SPAC977.04 |
|  | SPAPB1A10.08 |
|  | SPAC9E9.04 |
|  | SPAPB1E7.04c |
|  | SPAC16A10.01 |
|  | SPBC11G11.01 |
|  | SPBC1347.13c |
|  | SPAC6C3.02c |
|  | SPAC824.02 |
|  | SPAC823.03 |
|  | SPAC13C5.06c |
|  | SPBC685.10c |
|  | SPBC6B1.02 |
|  | SPAC19G12.09 |
|  | SPBC725.06c |
|  | SPBC4F6.16c |
|  | SPBC530.11c |
|  | SPBC577.08c |
|  | SPBC609.03 |
|  | nak1 |
|  | myo3 |
|  | SPBP8B7.24c |
|  | SPCC794.01c |
|  | SPAC3H1.08c |
|  | pcu3 |
|  | ntp1 |
|  | pyp1 |
|  | SPBC29A3.03c |
|  | SPBC29A3.10c |
|  | SPBC30D10.14 |
|  | SPAC13C5.01c |
|  | SPAC6F6.05 |
|  | SPBC27B12.03c |
|  | SPAC1834.09 |
|  | SPBC3H7.03c |
|  | SPBC354.11c |
|  | phz1 |
|  | SPBC947.09 |
|  | SPAC630.09c |
|  | SPAC5H10.02c |
|  | png2 |
|  | pi028 |
