# Supplementary material for: Genome-Wide Studies of Histone Demethylation Catalysed by the Fission Yeast Homologues of Mammalian LSD1
Source: PLoS One. 2007 Apr 18;2(4):e386. doi: 10.1371/journal.pone.0000386 (PMC1849891; doi:10.1371/journal.pone.0000386)
Supplement: Table S3 — Genes that have either increased levels of H3K4me2 or H3K9me2 in swm1 deletion cells (0.31 MB DOC) [file pone.0000386.s003.doc]

**Table S3** – Genes that have either increased levels of H3K4me2 or H3K9me2 in *swm1* deletion cells

(We used a cutoff value of >2 fold increase as compared to wild type to define IGR and ORF regions with increased H3K4me2 or H3K9me2 in at least 3 out of 4 experiments by the *swm1* deletion – see main text for further details).

| **HIGH IGR H3K4me2 in swm1 (3 of 4) 82 regions** | **HIGH ORF H3K4me2 in swm1 (3 of 4) 166 regions** | **HIGH IGR H3K9me2 in swm1 (3 of 4) 190 regions** | **HIGH ORF H3K9me2 in swm1 (3 of 4) 256 regions** |
| --- | --- | --- | --- |
|  |  |  |  |
| longIGRpuc1 | psu1 | sme1 | rad9 |
| p20C8_338912_1 | psk1 | scd1 | SPAC17H9.14c |
| longIGRptr2 | ptr2 | chc1 | rad17 |
| longIGRpma1 | pyk1 | coq5 | SPAC17G8.03c |
| p20C8_337239_1 | rhp4b | SPAC139.06 | rpb5 |
| SPCC1393.12 | SPCC16A11.08 | pop2 | rpl24-3 |
| longIGR | pcr1 | sid1 | SPCC16A11.03c |
| longIGRbgs4 | pfk1 | sec21 | SPCC16A11.12c |
| SPBPB2B2.18 | snu66 | rlc1 | SPCC1840.04 |
| 46/54/1.longIGRSPCC188.09c | SPAC1006.05c | bub1 | rhp4a |
| 34/54/1.longIGRSPCC794.04c | coq5 | B22918-1 | rnp24 |
| 9/54/1.longIGRSPAC6F6.13c | sec31 | mak1 | SPAC17A5.06 |
| 4/54/1.longIGRfin1 | prp1 | mip1 | ptb1 |
| longIGRmei2 | pob1 | alp4 | prs1 |
| longIGRctt1 | SPAC1006.06 | dld1 | pse1 |
| longIGRisp7 | prp16 | azr1 | pms1 |
| longIGRgit3R218 | SPCC306.08c | cap | pmc4 |
| mfm1 | SPBC9B6.09c | ecm2 | SPAC4G9.04c |
| mde7 | SPCC1919.05 | SPCC594.06c | pdh1 |
| agl | SPCC306.11 | SPBP4H10.16c | pdb1 |
| meu14 | SPBC8D2.01 | SPCC364.02c | pcu3 |
| SPAC29A4.12c | SPCC1259.08 | SPCC794.01c | SPAC630.07c |
| SPAC750.04c | SPBP35G2.11c | SPCC1827.03c | SPAC56F8.14c |
| exg2 | SPBP23A10.14c | longIGRfio1 | SPAC824.08 |
| SPAC4H3.03c | SPCC417.08 | SPBPB2B2.20c | SPBC8D2.12c |
| SPAC57A7.05 | SPCC4G3.12c | SPCC191.04c | SPCC126.06 |
| SPCC584.02 | SPCC364.04c | SPAPYUG7.07 | SPCC13B11.02c |
| SPCC306.11 | SPCC364.06 | rnh1 | SPBC83.15 |
| longIGRcps1 | SPCC4G3.01 | rfc3 | SPBC887.17 |
| SPCC1020.10 | SPCC4G3.07c | rpl37 | SPBC887.15c |
| rsv1 | SPCC4G3.14 | rps29 | SPBPB2B2.10c |
| cta3 | SPCC4G3.09c | SPCC553.06 | ubc1 |
| SPCC364.03 | SPAC664.15 | SPCC4G3.17 | vps17 |
| SPAC1348.03 | SPAC29B12.08 | rhp4b | SPBP8B7.27 |
| atf21 | SPAC17G6.11c | SPCC4B3.03c | SPBPB10D8.02c |
| longIGRSPAC1D4.11c | SPAC4G8.04 | SPCC24B10.20 | SPCC1020.10 |
| longIGRSPAC2C4.17c | SPAC29A4.11 | SPAC17C9.06 | SPAC186.03 |
| longIGRSPCC1020.10 | SPAC31G5.19 | SPAC17G6.01 | SPCC364.02c |
| longIGRSPAPB1A11.02 | SPAC27F1.08 | SPAC26A3.04 | SPCC306.08c |
| longIGRSPAC977.17 | SPAC29A4.12c | SPAC17C9.05c | SPCC1795.08c |
| longIGRSPAC9E9.09c | SPAC1D4.11c | SPAC1D4.02c | SPCC18.06c |
| longIGRSPBC9B6.13 | SPAC24C9.06c | SPAC1F7.03 | SPAC19B12.11c |
| longIGRSPBC354.09c | SPAC222.09 | SPAC17G6.07c | SPAC1851.04c |
| SPCP1E11.03 | SPAC1F3.01 | SPAC1782.02c | SPAC1851.02 |
| SPBC56F2.05c | SPAC13G6.10c | SPAC18B11.08c | SPAC18G6.11c |
| longIGRSPBP23A10.11c | SPAC186.07c | SPAC23C4.06c | SPCC4G3.11 |
| longIGRSPCC70.05c | SPAC23H4.15 | SPAC23C4.21 | SPCC4G3.09c |
| longIGRSPBC3H7.02 | SPAC25G10.09c | SPAC23A1.14c | gma12 |
| 25/54/1.longIGRcsx2 | cdc13 | SPAC23H3.11c | mus81 |
| 51/54/1.longIGRSPCC70.04c | ade3 | SPAC21E11.01 | orc5 |
| 12/54/1.longIGRagl | mex67 | SPAC22F3.11c | klp6 |
| 13/54/1.longIGRSPAC31G5.20c | bip | SPAC23C4.07 | kes1 |
| longIGRzfs1 | ef1-b | SPAC23G3.02c | fbp1 |
| 35/54/1.longIGRSPCC553.10 | cps1 | SPAC29B12.08 | map1 |
| telomericsimilartoc977 | aur1 | SPAC4G8.01c | SPAC6F6.03c |
| SPBC28E12.03 | cut6 | inv1 | SPAC688.07c |
| 7/54/1.longIGRSPAC6C3.01c | kes1 | SPAC3H8.03 | noc1 |
| SPBC1685.05 | kap123 | SPAC4G8.07c | nrd1 |
| int6 | SPAC328.02 | pac1 | meu26 |
| SPBC12C2.14c | hsp60 | par2 | SPAC323.04 |
| longIGRste7 | mus81 | isy1 | SPAC26A3.11 |
| vps3 | mhk1 | erg9 | SPAC27D7.08c |
| SPCC794.04c | fum1 | lsm6 | SPAC3G6.09c |
| SPAC56F8.15 | git3 | SPAC750.06c | SPAC3F10.07c |
| SPAC17A5.04c | SPCC1393.12 | SPAC977.12 | SPAC31G5.10 |
| SPAC26A3.17c | spp42 | SPAC18G6.13 | hmg1 |
| SPAC2F7.06c | SPCC622.15c | SPAC6B12.04c | his7 |
| SPBC609.01 | SPCC553.12c | SPAC3F10.05c | isp7 |
| longIGRSPBC1271.09 | SPBC1539.05 | SPAC3C7.09 | SPAC343.14c |
| longIGRSPAC57A7.05 | SPBC16E9.02c | SPAC9G1.08c | SPAC343.03 |
| pma2 | SPCC63.16 | SPAC8C9.12c | SPAC343.07 |
| SPAC1250.02 | SPBC4F6.12 | SPAC521.04c | SPAC12B10.16c |
| SPCC4G3.07c | SPCC663.15c | SPBC800.13 | SPAC4H3.04c |
| SPCC4G3.06c | sum3 | SPBC1734.01c | SPAC1039.05c |
| 5/54/1.longIGRSPAC5D6.04 | SPCC553.10 | SPBC1773.08c | rpn5-b |
| longIGRSPAPB2B4.04c | SPCC63.14 | SPBC16G5.06 | SPAC11E3.02c |
| ada1 | SPBC3H7.03c | SPBC8D2.21c | SPAC12G12.15 |
| longIGRSPBC32H8.02c | SPBC3D6.14c | SPBP16F5.09c | SPAC1002.10c |
| longIGRSPAC212.02 | SPBC1105.14 | uap56 | SPAC11D3.03c |
| longIGRSPCC320.03 | SPBC3D6.02 | SPCC965.02 | SPCC417.10 |
| SPCC1620.04c | SPBC3B9.16c | sum2 | SPAC4F10.09c |
| meu22 | SPBC660.12c | SPBC887.01 | spa2 |
|  | SPBC1778.10c | SPBC1773.04 | SPAC1002.19 |
|  | SPBC1539.04 | uch1 | abc1-x2 |
|  | SPBC354.13 | SPBC1289.15 | cut2 |
|  | SPAC9.09 | SPBC119.16c | SPAC6F6.09 |
|  | SPBC1861.10 | SPBC18H10.05 | cdb4 |
|  | SPBP8B7.15c | SPBC12C2.09c | SPAC821.05 |
|  | ulp1 | SPBC12C2.08 | cbh2 |
|  | vps9a | SPBC4B4.12c | cox10 |
|  | SPBP8B7.23 | SPBC530.04 | coq7 |
|  | ura1 | SPBC1709.12 | shm2 |
|  | wee1 | SPBC16D10.02 | din1 |
|  | Ef1-b dilution1x | SPBC18E5.14c | dsk1 |
|  | tif471 | SPBC21C3.12c | dak2 |
|  | trk2 | srw1 | SPAC25G10.03 |
|  | vps5 | SPBC25H2.14 | SPAC25B8.08 |
|  | SPBC27B12.11c | snw1 | SPBC3E7.04c |
|  | SPBC19F8.03c | tRNAPheanticodonGAA331228-331300 | SPBC1198.06c |
|  | tef1-e | 29/54/1.longIGRklp1 | SPAPB24D3.07c |
|  | SPBC25B2.12c | 21/54/1.longIGRSPBC2F12.12c | SPBC36.10 |
|  | SPAC1A6.07 | longIGRSPBC1271.09 | SPAC24B11.10c |
|  | SPAC806.02c | longIGRSPCC18B5.02c | SPAC1F7.09c |
|  | SPAC6G10.02c | longIGRSPCC1281.06c | SPAPJ691.03 |
|  | SPAC31G5.20c | SPBC3B8.07c | SPBC36B7.04 |
|  | SPBC31F10.16 | SPBC2F12.03c | SPBC3B8.04c |
|  | SPBC428.10 | SPBC16G5.17 | SPAC24H6.11c |
|  | SPBC409.08 | pB10D8_83590_1 | SPACUNK4.08 |
|  | SPBC4.02c | SPBC27B12.08 | SPAP8A3.06 |
|  | SPBC3B8.10c | ste13 | SPBC1734.06 |
|  | SPBC1A4.09 | SPAC3A11.09 | SPAC9E9.16 |
|  | SPAC6C3.01c | SPAC3A11.11c | SPAC8E11.01c |
|  | SPAC2F7.16c | SPBC577.09 | SPAC890.09 |
|  | SPAC1F7.03 | SPBC3B9.14c | SPAC922.06 |
|  | SPAC24B11.10c | SPAC343.15 | SPAC22F8.13 |
|  | SPAC1F3.03 | SPAC57A7.13 | SPAPB1A10.09 |
|  | SPBC713.13c | SPBC4F6.07c | SPAC8C9.05 |
|  | SPBC685.10c | SPBC3B9.08c | SPBC32F12.08c |
|  | SPAC23A1.17 | SPBC13A2.02 | SPAC212.03 |
|  | SPAC1565.08 | SPAC30D11.08c | SPBC317.01 |
|  | SPAC13G7.04c | SPAC2F7.07c | SPBC2D10.08c |
|  | SPAC25B8.11 | SPBC418.02 | SPBC2F12.15c |
|  | SPAC20G8.06 | SPAC323.01c | SPBC2A9.11c |
|  | SPAC17G8.11c | SPAC31G5.15 | SPAC20H4.09 |
|  | SPAC4G8.05 | SPAC1687.08 | SPBC29A10.10c |
|  | SPAC5D6.02c | SPBC2A9.07c | SPAC22E12.14c |
|  | SPAC17A2.05 | SPBC29A3.16 | SPBC27B12.05 |
|  | SPAC17A2.12 | SPAC26H5.09c | SPBC29A3.15c |
|  | SPAC20G8.08c | SPAC1B3.17 | SPBC6B1.03c |
|  | SPBC713.12 | SPAC29A4.15 | SPBC577.06c |
|  | rhp26 | SPBC30D10.09c | SPBC4F6.09 |
|  | rec10 | SPAC24C9.12c | SPBC660.17c |
|  | SPCC1620.11 | SPBC2A9.04c | SPBC660.16 |
|  | SPCC18.03 | SPBC29A3.05 | SPBC19G7.16 |
|  | SPCC70.05c | SPBC947.09 | SPBC30B4.02c |
|  | SPCC576.05 | SPBC9B6.11c | SPBC30B4.04c |
|  | pgi1 | SPBC25B2.07c | SPAC1D4.10 |
|  | phz1 | SPBC577.08c | SPBC428.17c |
|  | SPCC622.10c | SPBC27B12.03c | SPCC550.03c |
|  | vps27 | SPAC27F1.08 | SPAC13G7.06 |
|  | SPBC9B6.03 | SPAC20H4.02 | SPCC550.05 |
|  | SPCC1322.05c | SPBC31E1.05 | SPCC622.20c |
|  | SPCC1235.09 | SPBC24C6.08c | SPCC550.12 |
|  | Ef1-b dilution16x | SPBP35G2.12 | SPCC4G3.18 |
|  | Ef1-b dilution2x | SPAC22A12.10 | SPBC1685.14c |
|  | tpp1 | rng2 | SPBC16E9.09c |
|  | Ef1-b dilution8x | rpl32-2 | SPBC13E7.11 |
|  | vps53 | SPBC1773.03c | SPCP25A2.03 |
|  | SPBC1105.02c | SPCC4G3.06c | ste4 |
|  | SPAPJ696.02 | rpc11 | taz1 |
|  | SPAC824.08 | trx2 | sti1 |
|  | nda3 | rec11 | php5+ |
|  | SPBC3B9.19 | SPAC13G6.10c | vps16 |
|  | hmt1 | sec26 | ulp1 |
|  | itr1 | SPBC1734.09 | upf2 |
|  | itr2 | pi003 | SPAC13A11.04c |
|  | SPAC17A5.06 | SPCC1322.14c | SPCC622.10c |
|  | SPAC1250.01 | SPCC1223.04c | SPCC830.12 |
|  | SPCC895.08c | SPCC63.03 | rpn5 |
|  | SPAC1142.04 | c977_1926307_1 | SPAC14C4.09 |
|  | SPCC970.08 | longIGRSPAC750.01 | SPAC14C4.15c |
|  | SPBC17D1.08 | SPBPB2B2.04 | SPBC18E5.11c |
|  | cdc1 | SPCC297.04c | SPAC23C4.06c |
|  | SPBC146.13c | longIGRSPCC1393.07c | SPBC17D1.07c |
|  | SPBC17D11.08 | plc1 | SPAC1782.11 |
|  | SPBC1703.12 | SPCC576.18c | SPBC17A3.09c |
|  |  | longIGRzfs1 | SPBC17A3.08 |
|  |  | SPBC1105.08 | SPBC1773.17c |
|  |  | lsm7 | SPBC17A3.06 |
|  |  | SPAPB17E12.03 | SPBC21.03c |
|  |  | SPBC18E5.09c | SPAC16E8.04c |
|  |  | SPBC1105.07c | SPAC22G7.09c |
|  |  | mok14 | SPBC1709.14 |
|  |  | nrf1 | SPAC16E8.06c |
|  |  | SPBC16A3.19 | SPBC16G5.05c |
|  |  | SPBC1683.06c | SPBC18E5.09c |
|  |  | mde2 | SPAC24C9.14 |
|  |  | SPBC1604.04 | SPAC29B12.05c |
|  |  | sfc1 | SPAC144.15c |
|  |  | sec61 | SPAC144.05 |
|  |  | SPBC17A3.06 | SPAC23E2.02 |
|  |  | SPCC320.04c | SPAC18G6.09c |
|  |  | sds23 | SPAC1952.15c |
|  |  | sft1 | SPAC6F6.10c |
|  |  | act1 | SPAC24H6.02c |
|  |  | cal1 | SPAC6C3.10c |
|  |  | pep7 | SPAC23G3.09 |
|  |  | SPAC1142.04 | SPAC13G7.04c |
|  |  | SPBC16D10.08c | SPAC25B8.12c |
|  |  | dmf1 | SPAC22F3.06c |
|  |  |  | SPAC22F3.07c |
|  |  |  | SPAC6F12.06 |
|  |  |  | SPAC26H5.05 |
|  |  |  | SPAC25B8.06c |
|  |  |  | SPAC1D4.08 |
|  |  |  | SPAC1B3.15c |
|  |  |  | SPAC9G1.05 |
|  |  |  | SPBC1734.01c |
|  |  |  | SPBC18E5.05c |
|  |  |  | SPBC17G9.03c |
|  |  |  | SPBC119.03 |
|  |  |  | SPAPB21F2.02 |
|  |  |  | SPAC9.07c |
|  |  |  | SPBC16E9.10c |
|  |  |  | SPCC550.01c |
|  |  |  | zpr1 |
|  |  |  | vps3 |
|  |  |  | SPAC9G1.12 |
|  |  |  | SPBC14C8.15 |
|  |  |  | SPBC14F5.06 |
|  |  |  | SPBC15C4.06c |
|  |  |  | SPBC19C7.12c |
|  |  |  | SPBC19C2.12 |
|  |  |  | SPBC25B2.06c |
|  |  |  | SPBC21B10.08c |
|  |  |  | SPBC28E12.06c |
|  |  |  | SPBC31F10.14c |
|  |  |  | SPBC31F10.03 |
|  |  |  | SPBC342.04 |
|  |  |  | SPBC3E7.10 |
|  |  |  | SPBC106.13 |
|  |  |  | SPBC106.14c |
|  |  |  | SPBC21.02 |
|  |  |  | SPBC660.12c |
|  |  |  | SPBC577.12 |
|  |  |  | SPBC651.12c |
|  |  |  | rps11-2 |
|  |  |  | dis3 |
|  |  |  | but1 |
|  |  |  | cdc1 |
|  |  |  | pef1 |
|  |  |  | pof13 |
|  |  |  | SPAC1002.06c |
|  |  |  | ipk1 |
|  |  |  | SPAC31A2.02 |
|  |  |  | SPAC323.03c |
|  |  |  | SPAC22A12.10 |
|  |  |  | SPAC12G12.06c |
|  |  |  | B13958-1 |
|  |  |  | B20341-2 |
|  |  |  | idi1 |
|  |  |  | SPBP35G2.12 |
|  |  |  | SPBC947.08c |
|  |  |  | SPBC947.10 |
|  |  |  | SPCC364.06 |
|  |  |  | eft2 |
|  |  |  | tor2 |
|  |  |  | top2 |
|  |  |  | rds1 |
|  |  |  | pi028 |
|  |  |  | pch1 |
|  |  |  | pck1 |
|  |  |  | SPBC8D2.01 |
|  |  |  | SPCC1620.06c |
|  |  |  | SPCC1739.06c |
|  |  |  | rec8 |
